# Identification of Autoimmunity to Peptides of Collagen V α1 Chain as Newly Biomarkers of Early Stage of Systemic Sclerosis

**DOI:** 10.3389/fimmu.2020.604602

**Published:** 2021-02-12

**Authors:** Ana Paula Pereira Velosa, Lais Brito, Zelita Aparecida de Jesus Queiroz, Solange Carrasco, Jurandir Tomaz de Miranda, Cecília Farhat, Cláudia Goldenstein-Schainberg, Edwin Roger Parra, Danieli Castro Oliveira de Andrade, Pedro Leme Silva, Vera Luiza Capelozzi, Walcy Rosolia Teodoro

**Affiliations:** ^1^ Rheumatology Division of the Hospital das Clinicas FMUSP, Faculdade de Medicina, Universidade de Sao Paulo, Sao Paulo, Brazil; ^2^ Department of Pathology of the Faculdade de Medicina, Universidade de Sao Paulo, Sao Paulo, Brazil; ^3^ Laboratory of Pulmonary Investigation, Carlos Chagas Filho Biophysics Institute, Federal University of Rio de Janeiro, Centro de Ciências da Saúde, Rio de Janeiro, Brazil; ^4^ National Institute of Science and Technology for Regenerative Medicine, Rio de Janeiro, Brazil

**Keywords:** type V collagen, autoimmunity, early-SSc, α1(V) chain, biomarker

## Abstract

Patients with Systemic sclerosis (SSc) presents immune dysregulation, vasculopathy, and fibrosis of the skin and various internal organs. Pulmonary fibrosis leads to SSc-associated interstitial lung disease (ILD), which is the main cause of morbidity and mortality in SSc. Recently autoimmunity to type V collagen (Col V) has been characterized in idiopathic pulmonary fibrosis and show promise to be related to the development in SSc. Our aim was to evaluate autoimmunity to Col V α1(V) and α2(V) chains and to the antigenic peptides of these Col V chains in early-SSc sera employing lung tissue of SSc-ILD, as antigen source. We found that sera samples from patients with early-SSc were reactive to Col V (41.18%) and presented immunoreactivity for Col5A1(1.049) and Col5A1(1.439) peptides. The IgG isolated from early-SSc patients-anti-Col V positive sera (anti-ColV IgG) was adsorbed with α1(V) chain (anti-ColV IgG/ads-α1(V)) and α2(V) chain (anti-ColV IgG/ads-α2(V)) and biotinylated to evaluate the spectrum of reactivity in SSc-ILD patients lung biopsies by immunofluorescence. The SSc-ILD lung tissue samples immunostained with anti-ColV IgG showed increased green fluorescence in the vascular basement membrane, bronchiolar smooth muscle, and adventitial layer, contrasting with the tenue immunostaining in control lungs. Col V protein expression in these pulmonary compartments immunostained with early-SSc anti-ColV IgG was confirmed by immune colocalization assays with commercial anti-human Col V antibodies. In addition, SSc-ILD lung tissues immunostained with anti-ColV IgG/ads-α1(V) (sample in which Col V α1 chain-specific antibodies were removed) showed decreased green fluorescence compared to anti-ColV IgG and anti-ColV IgG/ads-α2(V). Our data show that autoimmunity to Col V in early-SSc was related to peptides of the α1(V) chain, suggesting that these antibodies could be biomarkers of SSc stages and potential target of immunotherapy with Col V immunogenic peptides.

## Introduction

Systemic sclerosis (SSc) is an autoimmune disease that belongs to the family of systemic connective tissue diseases characterized by immune dysregulation, vasculopathy, and fibrosis of the skin and various internal organs. An excessive expression of proteins in the extracellular matrix (ECM) leads to progressive organ stiffening, which is a hallmark of these diseases. Fibrosis causes SSc-associated interstitial lung disease (ILD), which leads to high morbidity and mortality in SSc (1, 2).

The ECM contains a significant number of “informational” proteins, glycoproteins, proteoglycans and polysaccharides whose interactions with epithelial and endothelial cells play a major role in the control of cell migration, cell adhesion, angiogenesis, and tissue development and repair (3). One of the commonest features in SSc is a massive exposure of the epithelial/endothelial basement membrane to autoimmune cells. The literature also describes marked qualitative and quantitative modifications of the interstitial collagen in SSc patients (4, 5).

Collagen V (Col V) is a minor component of the ECM with peculiar biochemical and immunologic characteristics. This protein is highly preserved, highly immunogenic and maintains the NH2 terminal region during its biosynthesis (6–8). In the pulmonary tissue, Col V is located in the interstitium and capillary basement membranes and is surrounded by vascular smooth cells (9). The Col V isoform [α1(V)_2_, α2(V)] is ubiquitous in tissues and contains a fibrillary portion embedded within the heterotypic collagen I/III fibrils. Consequently, it is considered a sequestered antigen and can become an autoantigen when exposed to the immune system as a result of chronic tissue remodeling processes (6, 10–14). Autoimmunity to Col V has been associated with several pathologies, such as rejection of lung grafts in experimental models and humans, idiopathic pulmonary fibrosis, hypersensitivity asthma, and atherosclerosis (8, 15–19). In relation to SSc, several clinical studies have shown that patients present an increased expression of Col V in the skin and lung (20–23). Furthermore, in the earliest stages of the disease, increased Col V in the skin was correlated with cutaneous thickening and disease activity (24). Of note among the range of autoantibodies characteristic of SSc, anti-Col V antibodies were present in about 31% of the patients (25, 26). Yet, a recent study described anti-Col V antibodies in 33% of the patients in the early stage of SSc (27). In addition to clinical findings, our group described a preclinical model for SSc using Col V immunization, which leads to histological, vascular and immunological changes similar to those observed in human SSc (28–30). In this SSc experimental model, Col V-induced nasal tolerance also modulated the reversion of inflammation and fibrosis in the lung and skin, reinforcing the role of Col V in the pathogenesis of SSc as one of the neoantigens triggering autoimmune response (31, 32).

Certain immunopathogenic Col V peptides appear to be present in different diseases and pathologic processes. For instance, in atherosclerosis, studies have shown autoimmunity to two peptides of the α1(V) chain [Col5A1(599) and Col5A1(909)] (33). Similarly, in lung transplantation, an immunodominance of self-antigenic determinants to specific α1(V) chain peptides resulted in loss of tolerance leading to autoimmunity to Col V and chronic lung allograft rejection (34, 35). However, the pathogenic epitopes of Col V associated with patient susceptibility to Col V autoimmunity in SSc remain unknown. The question also remains as to whether Col V plays a role in *de novo* autoimmunity.

Despite the advances in the treatment of SSc with immunosuppressors and antifibrotic drugs in the past years, the death rate from SSc remains largely unchanged and mainly caused by ILD (36–38). SSc-ILD is the phenotypic consequence of the interactions between the cellular and extracellular matrices resulted from epithelial/endothelial injury that lead to both the transformation of fibroblasts into a myofibroblastic phenotype and an excess deposition of collagen fibers (5). There is therefore great interest in finding the means to identify which mechanistic processes are involved in SSc-ILD and to improve therapeutic efforts so as to optimize the quality of life for each patient.

In the light of previous data suggesting autoimmunity to peptides of the α1(V) chain in other diseases, the present study aims to (1) evaluate autoimmunity to α1(V) and α2(V) chains in SSc based on the antigenicity of sera from early-SSc patients and on lung tissue specimens from SSc patients with ILD (2) determine the autoimmunity to five α1(V) chain peptides and three α2(V) chain antigenic peptides in sera of early-SSc patients (33–35).

We found that the reactivity of the early-SSc patients’ sera with the SSc-ILD lung biopsies related strongly to autoimmunity to α1(V) chain in of SSc, specially to two immunogenic α1(V) chain peptides. Therefore, our findings suggest these autoantibodies could be biomarkers of early-SSc phase. Furthermore, the immunotherapy with Col V immunogenic peptides could be a promising mark for new therapeutic approaches.

## Materials and Methods

### Patients and Samples

Frozen sera samples, stored in the biorepository of the Department of Rheumatology of the University of São Paulo Medical School, from 17 patients with early-SSc involved in previous studies (27), were included in the study. Patients presenting puffy fingers, Raynaud’s phenomenon, abnormal capillaroscopy with scleroderma pattern, antinuclear, anticentromere and antitopoisomerase-I antibodies were categorized as early-SSc (EULAR Preliminary Criteria) (39, 40). **Supplementary Table 1** summarizes clinical and laboratorial data from early-SSc patients.

Between January 2002 and July 2004, 28 consecutive patients with defined SSc-ILD, presenting associated respiratory symptoms and/or reduced performance on a pulmonary function test underwent an open lung biopsy and were followed for at least 3 years. We recovered 4 formalin fixed paraffin embedded from patient involved in previous studies (41, 42) (24 samples were not included due to wear lack of lung material). These patients included in the present study were classified as having diffuse SSc (dSSc) (n =2) and limited SSc (lcSSc) (n =2), according to diagnostic (Preliminary criteria for the classification of systemic sclerosis (scleroderma) (43) and subtype criteria (44). These formalin fixed paraffin embedded lung tissue samples obtained by surgical biopsy from these 4 patients with SSc-ILD archived in Department of Pathology of our institution were included in the study.

Frozen sera samples, stored in the biorepository of our Department of Rheumatology, from healthy age- and sex-matched volunteers were included as control. Formalin fixed paraffin embedded normal lungs achieved in our Department of Pathology were obtained from autopsies of age and sex-matched patients who died due to brain aneurism or hemorrhagic stroke due to systemic arterial hypertension.

The lung samples access was approved by Research Ethics Committee (CAPPesq No. 0960/08) at the Hospital das Clínicas of the University of São Paulo Medical School. The sera samples access was approved by Research Ethics Committee (CAPPesq No. 675.003/2014) at the Hospital das Clínicas of the University of São Paulo Medical School.

## Detection of Autoantibodies to Col V and Col V Peptides in Early-SSc

### Flow Cytometry Assay

A flow cytometry bead assay detected antibodies to Col V and Col V synthetic peptides in early-SSc sera samples, using the method described by Iwata et al. (45), with modifications. Before the cytometry, Col V from bovine placenta (Sigma chemical co., St. Louis, Missouri), which is 97.13–99.31% homologous to human Col V, and 15-mer Col V peptides (**Table 1**) had been bound to biotin with the EZ-Link^®^ Sulfo-NHS-Biotinylation kit (Thermo Scientific/Pierce, Rockford, Illinois) in accordance with the manufacturer’s instructions. After that, 1x10^7^ streptavidin microspheres (Streptavidin Microspheres, 6.0 Micron; Polysciences, Inc, Warrington, Pennsylvania) were sensitized with biotinylated bovine placental Col V (50 μg/mL) and Col V peptides (150 μg/mL) in binding buffer (Phosphate 0.1 M, pH 7.4 with NaCl 0,9% and BSA1%) for 60 min at 4°C. The sensitized microspheres were maintained at 4°C until the procedures. In the flow cytometry, 17 early-SSc sera samples were screened to detect anti-ColV positive antibodies. For this, both early-SSc and control (n=6) sera samples were incubated with 1x10^6^ Col V-sensitized streptavidin microspheres in binding buffer for 30 min at room temperature. The microspheres were then washed in Phosphate buffer at 1,500 rpm centrifugation during 5 min and incubated with ALEXA FLUOR 488 anti-human IgG conjugate (1:250; Invitrogen Life Technologies, Eugene, Oregon) in the dark during 30 min at room temperature. After a second washing cycle, the assays were then analyzed in a Flow Cytometer (FACSCalibur Becton-Dickson, San Jose, California). For the next study stage, we considered the percentage of early-SSc sera samples, tested positive to anti-Col V autoantibodies.

**Table 1:** The amino acids sequence of the immunogenic Col V peptides.

Peptides	Amino acids sequence	References
Col5A1(599)	PPGPAGKPGRRG	Park et al. (33)
Col5A1(779)	GIRGLKGTKGEKGED	Keller et al. (35)
Col5A1(909)	RGQRGPTGPRGERGPRG	Park et al. (33)
Col5A1(1049)	KDGPPGLRGFPGDRG	Keller et al. (35)
Col5A1(1439)	LRGIPGPVGEQGLPG	Keller et al. (35)
Col5A2(275)	PGEVGFAGSPFARGF	Tiriveedhi et al. (34)
Col5A2(419)	PGAIGTDGTPGAKGP	Tiriveedhi et al. (34)
Col5A2(1078)	NIRFRYIVLQDTCSK	Tiriveedhi et al. (34)

Col5A1, Col V α1 chain peptide; Col5A2, Col V α2 chain peptide.

To evaluate autoantibodies to Col V immunogenic peptides, both the early-SSc sera samples positive to anti-Col V and the control sera samples were incubated with 3x10^6^ streptavidin microspheres sensitized with Col V peptides (**Table 1**) following the same procedures. The results were given as percentage of the fluorescent bead-bonded anti-Col V peptides in sera from early-SSc patients and healthy individuals quantified by flow cytometry.

### Immunogenic Col V Peptides

The 15-mer Col V peptides used in this study were selected based on their antigenicity and role in the autoimmunity of other diseases and pathologic processes (33–35). All five α1(V) chain peptides (Col5A1(599); Col5A1(779); Col5A1(909); Col5A1(1049) e Col5A1(1439)) and three α2(V) chain peptides [Col5A2(275); Col5A2(419) e Col5A2(1078)] selected were synthesized by GenScript^®^ (Piscataway, New Jersey) (**Table 1**).

### Isolation of α1(V) and α2(V) Chains

An affinity chromatography with a heparin column (HiTrap™ Heparin HP 1mL, GE Healthcare, Uppsala, Sweden) was employed to isolate the α1(V) chain from the commercial human placenta Col V (Sigma Chemical Co., St. Louis, Missouri). Col V was diluted in 0.01N acetic acid and dialyzed with 10 mM sodium phosphate, 0.15 M NaCl, pH 7.4. After 24 h of dialysis, all Col V samples were denatured at 50°C for 15 min to separate the α chains and then applied to the heparin column, which had been previously equilibrated with a 10 mM phosphate buffer/0.15 M NaCl, pH 7.4, at a rate of 0.5 mL/min. Under these conditions, the α1(V) chain binds to the heparin and α2(V)/α3(V) chains are collected in the eluate flow. The α1(V) chain was eluted from the heparin column with 10 mM sodium phosphate/1.5 M NaCl buffer, pH 7.4. First, the eluted Col V α chains samples were submitted to reading in a spectrophotometer at 230 nm. Then, the α2(V)/α3(V) eluate was submitted to polyacrylamide gel 6% electrophoresis (SDS-PAGE) and the α2(V) chain was electroeluted from the gel in Electro-Eluter (Bio-Rad, Hercules, California), according to the manufacturer’s instructions.

### Evaluation of the Immunogenicity to α1(V) and α2(V) Chains in SSc

#### Adsorption of IgG Fraction From Early-SSc Sera Tested Positive to Anti-ColV

Three sera samples tested positive to anti-Col V antibodies, previously determined by the flow cytometry, were pooled and their IgG fractions (anti-ColV IgG) were isolated by affinity chromatography, with HiTrap Protein A (GE Healthcare, Uppsala, Sweden). The anti-ColV IgG fraction was subjected to further biotinylation employing the EZ-Link^®^ Sulfo-NHS-Biotinylation Kit (Thermo scientific/Pierce, Rockford, Illinois), according to the instructions of the manufacturer.

For specific anti-Col V α1 and α2 chains adsorption, 96-well polystyrene plates were initially sensitized with 5 μg/well of isolated α1(V) and α2(V) chains in bicarbonate buffer at pH 9.6 for 16 h at 4°C. After the plates were blocked for 90 min with BSA 1% in PBS with 0.05% Tween20, and incubated for 2h30 at room temperature with the biotinylated anti-ColV IgG from early-SSc samples. The resultant samples were identified as biotinylated anti-ColV IgG adsorbed with α1(V) (anti-ColV IgG/ads-α1(V); Col V α1 chain-specific antibodies were removed from this sample) and α2(V) (anti-ColV IgG/ads-α2(V); Col V α2 chain-specific antibodies were removed from this sample) chains.

#### Immunofluorescence and Morphometric Analysis

For the immunofluorescence, slides with 4 to 5 μm of lung biopsies from SSc-ILD samples fixed in 10% formalin and embedded in paraffin were deparaffinized and treated with 8 mg/mL pepsin (Sigma Chemical Co, St. Louis, Missouri) diluted in acetic acid 0.5 N for 30 min at 37°C to expose the immunogenic sites. After blocking with 5% BSA in PBS, sections were incubated overnight at 4°C with biotinylated anti-ColV IgG (1:20), anti-ColV IgG/ads-α1(V) (1:20) and anti-ColV IgG/ads-α2(V) (1:20) diluted in PBS. The reaction was revealed by the incubation of ALEXA Fluor 488 streptavidin (Invitrogen Life Technologies, Eugene, Oregon), diluted 1:100 in 0.006% Evans blue in PBS, and visualized on an Olympus BX-51 (Tokio, Japan) fluorescence microscope.

The lung biopsies immunostained with anti-ColV IgG, anti-ColV IgG/ads-α1(V) and anti-ColV IgG/ads-α2(V) underwent a morphometric analysis on an *Image-ProPlus 6.0* system composed of an Olympus camera (Olympus Co, St Laurent, Quebec, Canada) coupled to an Olympus microscope (Olympus BX-51, Tokio, Japan). A digitizing system (Oculus TCX, Coreco, Inc., St. Laurent, Quebec, Canada) sent the images to an LG monitor, which were then downloaded to a computer (Pentium 1330 Mhz). The fluorescent area in the SSc-ILD pulmonary tissue was determined at 400× magnification in 10 random fields, adjusting the threshold level of measurement up to the density of immunostaining (fluorescent green). The results were calculated dividing the total area of immunostaining by the total area of the tissue and expressed as a percentage.

To evaluate the binding specificity of the anti-ColV IgG antibodies from early-SSc to Col V in the SSc-ILD lung biopsies, we employed double staining methods to assess colocalization. Again, after blocking with 5% BSA in PBS, sections of the lung biopsies from SSc-ILD were incubated simultaneously with rabbit anti-human Col V (1:100; Rockland Inc., Limerick, Pennsylvania) and early-SSc biotinylated anti-ColV IgG (1:20) overnight at 4°C. After incubation with ALEXA Fluor 488 streptavidin (1:100; Invitrogen Life Technologies, Eugene, Oregon), anti-rabbit ALEXA Fluor 546 (1:200; Invitrogen Life Technologies, Eugene, Oregon) and DAPI (1:200; Molecular Probes, Eugene, Oregon), the immunostaining was captured on an Olympus BX-51 (Tokio, Japan) fluorescence microscope. The images were then colocalized using the *software Image J 5.1.*

#### Statistical Analysis

Descriptive statistics was used to characterize the cohort and samples with determination of frequencies, means, medians, standard deviation, standard error and measures of central dispersion. The median value was used to dichotomize series of continuous variables when needed. Continuous variables presenting non-parametric data distribution were compared by Mann-Whitney test and independent-samples Kruskal-Wallis test, Continuous variables presenting parametric data distribution were compared by Student t-test test. Categorical variables were compared by Fisher’s exact test. The statistical software programs IBM SPSS (version 22; Armonk, NY, USA; RRID : SCR_002865) and S-Plus (version 8.04; TIBCO, Palo Alto, CA, USA) were used to perform the computations for all analyses. P-value<0.05 was considered significant.

## Results

**Figure 1:**
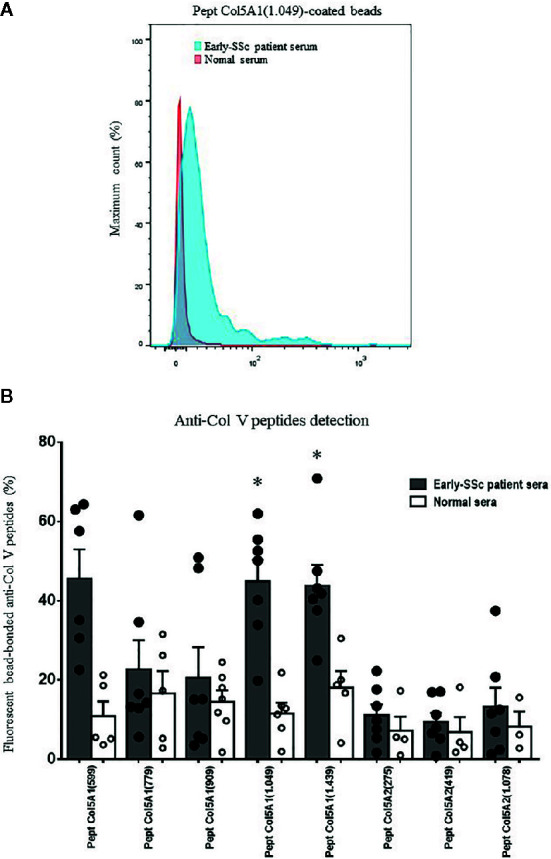
Detection of systemic antibodies to α1(V) and α2(V) peptides. The early-SSc patient and normal sera were incubated with streptavidin beads coated with Col5A1(599), Col5A1(779), Col5A1(909), Col5A1(1049), Col5A1(1439), Col5A2(275), Col5A2(419) and Col5A2(1078) peptides. **(A)** Flow cytometry was used to detect IgG antibodies bound to Col V peptides. Data shown are derived from serum collected from an early-SSc patient and a normal individual and are representative of n=7 patients sera tested positive to anti-Col V and n=6 normal sera. **(B)** Graphic of the anti-Col V peptides detection by flow cytometry, expressed as percentage of the fluorescent bead-bonded anti-Col V peptides of the α1(V) chain and α2(V) chain in sera from early-SSc patients and normal individuals. The filled points and the empty points represent the individual values of the detection of anti-Col V peptide antibodies, respectively, in the serum of patients and controls. Among the eight tested peptides, the Pept Col5.1 (1049) and Pept Col5.1 (1439) were more expressed in early-SSc sera in relation to control. (Student t test; *P < 0.05).

**Figure 2:**
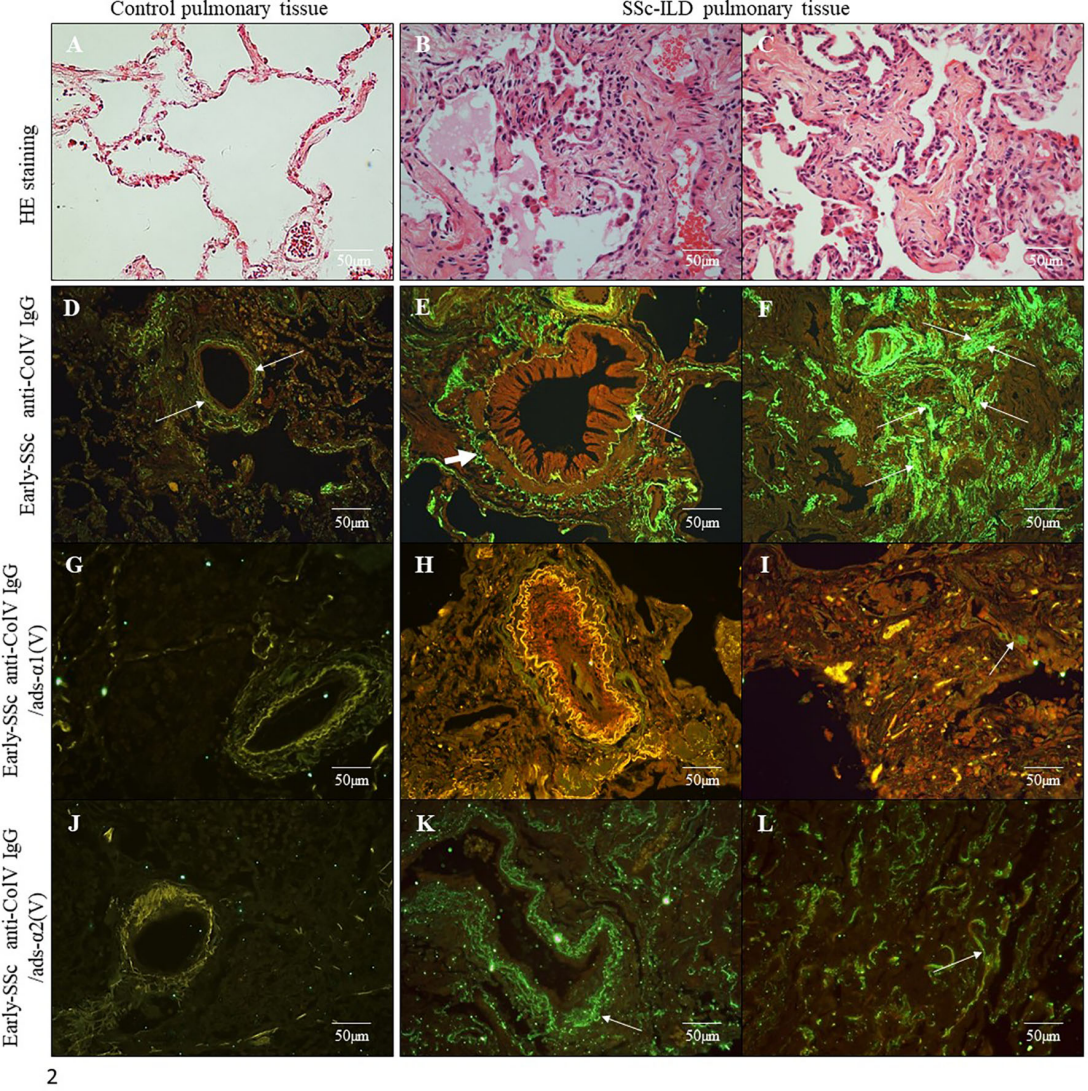
Histological sections of lung samples from control subjects without associated pulmonary pathology **(A)** and from SSc-ILD patients with a histological pattern of Non-Specific Interstitial Pneumonia (NSIP) **(B, C)** (Hematoxylin & Eosin staining; original magnification: **A–C**, X400). Immunofluorescence in lung sections from control samples **(D, G, J)** and SSc-ILD patients **(E, F, H, I, K, L)** immunostained with early-SSc biotinylated anti-ColV IgG **(D–F)**, anti-ColV IgG/ads-α1(V) **(G–I)** and anti-ColV IgG/ads-α2(V) **(J–L)**. Note the green immunofluorescence along of periadventitial layer of the bronchovascular axis **(E, K)** and alveolar septa **(F, L)** layers (arrows). The reaction was reveled with ALEXA Fluor 488 streptavidin. Original magnification: 400X, **(D–L)**.

We first submitted all 17 early-SSc sera samples to a flow cytometry assay to detect anti-ColV antibodies, of which 7 (41.18%) were positive. The anti-Col V presence or absence had no association with clinical features or SSc antibodies specificities, but anti-Col V presence was associated with the time of the first disease symptom (p=0.008) (**Supplementary Table 1**). **Figure 1A** shows the flow cytometry analysis from an early-SSc patient serum and a normal serum with detected IgG antibodies bound to Col5A1(1049) peptide. **Figure 1B** shows autoantibodies positivity in sera samples tested positive to anti-Col V to the Col V peptides (**Table 1**), already identified as triggers of autoimmunity in other pathologies (33–35). The flow cytometry assay demonstrated that a significant percentage of early-SSc sera samples positive to anti-ColV reacted to the Col5A1(1049) (p=0.001) and Col5A1(1439) (p=0.009) peptides of the α1(V) chain (**Figure 1** **;** **Supplementary Table 2**). **Figure 2** shows the histologic pattern of the pulmonary tissue from control subjects and SSc-ILD patients. While control lungs have a thin alveolar septa covered by a single layer of alveolar epithelium, characteristic of the normal histoarchitecture of the lung (**Figure 2A**), SSc-ILD lung biopsy tissue exhibits a homogeneous septal thickening through inflammation and fibrosis with the maintenance of the lung histoarchitecture, thus characterizing a nonspecific interstitial pneumonia (NSIP) histologic pattern (**Figures 2B, C**). The bronchovascular axis from each specimen showed hypertrophy of the smooth muscle and fibrosis of the adventitia layers (**Figure 2B**).

**Figure 3:**
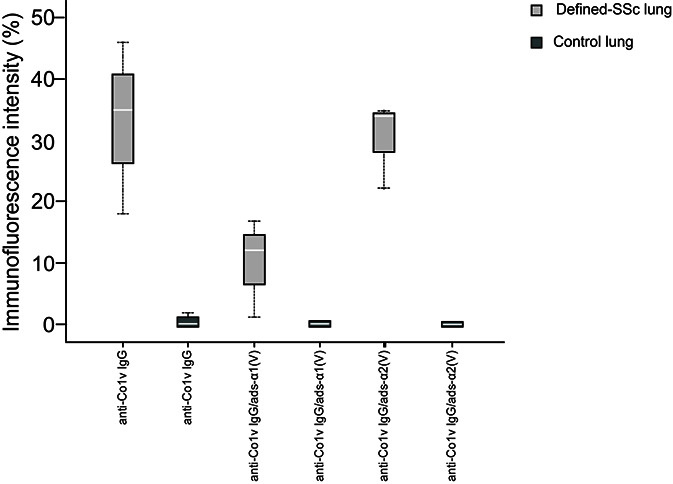
Box plot graphs of the immunofluorescence staining quantification of biotinylated early-SSc anti-ColV IgG, anti-ColV IgG/ads-α1(V) and anti-ColV IgG/ads-α2(V) detection, expressed as percentage, in lung tissue from defined-SSc patients and normal lungs. Each box corresponds to individual immunofluorescence intensity and lung tissue in defined-SSc patients and normal lungs. The solid bar represents the values of anti-Col V α1 or α2 chains between the 25th and 75th percentiles, the white bar shows the median value, and the top and bottom brackets show the extreme values whereas black dotted lines indicate the median anti-Col V level. *P<0.05 (Independent- Samples Kruskall-Wallis test) comparing median biotinylated early-SSc anti-ColV IgG in defined-SSc vs normal lung (33.00 ± 14.10 vs 0.20 ± 0.44) and median biotinylated early-SSc anti-ColV IgG vs early-SSc anti-ColV IgG/ads-α1(V) in defined-SSc vs normal lung (33.00 ± 14.10 vs 10.00 ± 8.18). Each group n=7. SSc, Systemic sclerosis; ads, adsorbed.

In order to evaluate the autoimmune potential of Col V α1(V) and α2(V) chains in SSc, the anti-ColV IgG, anti-ColV IgG/ads-α1(V) and anti-ColV IgG/ads-α2(V) samples from early-SSc were then employed in the immunostaining of both control and SSc-ILD lung tissue biopsies. **Figures 2D–L** show the distribution and characteristics of anti-ColV IgG, anti-ColV IgG/ads-α1(V) and anti-ColV IgG/ads-α2(V) immunostaining in control and SSc-ILD lung tissue biopsies. When immunostained with anti-ColV IgG, the biopsies of the control lung tissue showed a weak green fluorescence in a fine and homogenous pattern of the fibers surrounding the bronchovascular axis and along of the alveolar septa (**Figure 2D**). In contrast, the SSc-ILD lung tissue biopsies showed an increased green fluorescence, with a linear pattern of the fibers surrounding the layers of the bronchovascular axis (**Figure 2E**) and alveolar septa (**Figure 2F**). The SSc-ILD bronchioles show a particularly intense green fluorescence of the fibers along the basement membrane (**Figure 2E**, thin arrow) and adventitia layers (**Figure 2E**, thick arrow). We also noted a marked green fluorescence, with a thin, heterogeneous, and fine network pattern of the fibers between the smooth muscle and the periadventitial layers of the bronchoalveolar axis (**Figure 2E**), which coincides with the usual location of Col V fibers in this tissue. Moreover, the alveolar septa in SSc-ILD samples exhibited a strong green immunofluorescence, coinciding with a significant increase in thick Col V fibers. When compared to the control samples, these fibers assumed an irregular and micronodular distribution involving the septal interstitium and smaller vessels (**Figure 2F**). Histomorphometric analysis showed a significant increased immunostaining in SSc-ILD lung tissue biopsies incubated with early-SSc anti-ColV IgG compared to control lungs (p=0.009) (**Figure 3**). Conversely, when immunostained with anti-ColV IgG/ads-α1(V), the SSc-ILD lung biopsies exhibited a tenue green fluorescence of the smooth muscle and periadventitial fibers surrounding the bronchovascular axis and along the alveolar septa (**Figures 2H, I**). Similar results were found in the control lung tissue (**Figure 2G**). The histomorphometric analysis showed a significant decrease in the immunostaining of anti-ColV IgG/ads-α1(V) compared to anti-ColV IgG (p=0.018) (**Figure 3**). These results contrast with the immunofluorescence pattern from the anti-ColV IgG/ads-α2(V) staining, in which the SSc-ILD lung biopsies exhibited a strong green fluorescence around the bronchovascular axis and along of the thickened alveolar septa, whereas the expression in the control lung was weak (**Figures 2J–L**, **Figure 3**). Interestingly, when the anti-ColV IgG/ads-α2(V) was incubated with SSc-ILD lungs the green fluorescence of the fibers in the alveolar septa (**Figures 2K, L**) mostly coincides with the standard pattern found in anti-ColV IgG labeling (**Figures 2E, F**). In the same way, histomorphometric analysis showed no significant difference in immunostaining quantification in both anti-ColV IgG and anti-ColV IgG/ads-α2(V) (**Figure 3**).

**Figure 4:**
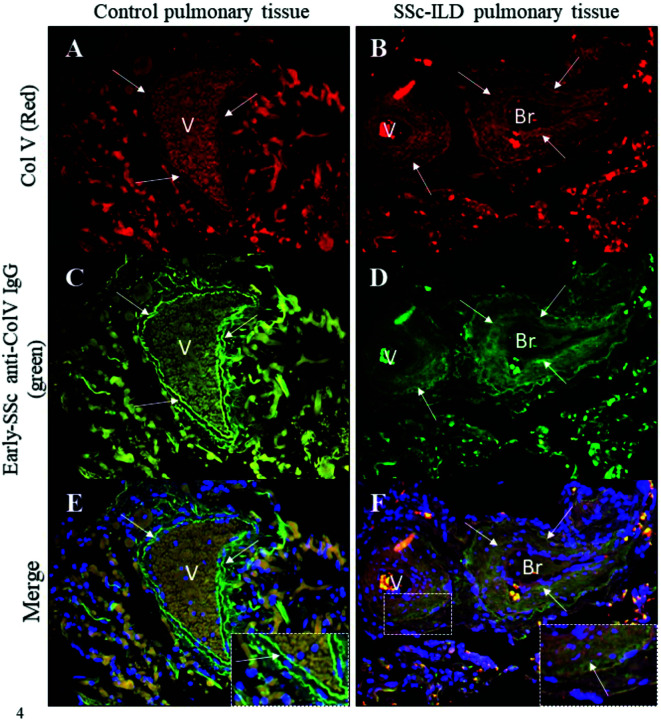
Immunofluorescence in lung sections from control and SSc-ILD patients’ pulmonary tissues shows Col V immunostained in red (**A, B**, arrows) and the immunolabeling of biotinylated anti-ColV IgG from early-SSc immunostained in green (**C**, **D**, arrows). The merged images show the colocalization of Col V and early-SSc anti-ColV IgG in the vessels and bronchiole basal layers and along the alveolar septa in SSc-ILD pulmonary tissue as seen in a yellowish green immunostaining (**F**, arrows). The nuclei are stained in blue **(E, F)**. Note, in the detail, the colocalization of Col V and early-SSc anti-ColV IgG in yellowish green fluorescence in the vessel layer from a SSc-ILD tissue sample **(F)**. Original magnification: 400X, **(A–H)**.

**Figure 4** shows the colocalized fluorescence between the anti-ColV IgG that had been isolated from the early-SSc sera samples that tested positive to anti-Col V autoantibodies and a commercial anti-human Col V antibody (Sigma Chemical) in both SSc-ILD and control lung biopsies. Lung tissue from control individuals and SSc-ILD patients exhibited the immunostained Col V in red (**Figures 4A, B**) and biotinylated early-SSc anti-ColV IgG in green (**Figures 4C, D**). The strong yellowish green fluorescence in the merged images of SSc-ILD lung biopsies indicates the colocalization of early-SSc anti-ColV IgG and tissue Col V around the basement membrane, smooth muscle and adventitial layers of the bronchovascular axis and along the alveolar septa (**Figure 4F**). A similar weak yellowish green fluorescence indicates this colocalization of early-SSc anti-ColV IgG and tissue Col V in control lung biopsies as well (**Figure 4E**). The nuclei are stained in blue (**Figures 4E, F**).

Considering that our data showed the same pattern of immunostaining in the SSc-ILD lung biopsies, incubated with anti-ColV IgG without previous absorption and after absorption with α2(V) chain (anti-ColV IgG/ads-α2(V)), we evaluated a possible correlation, between SSc-ILD lung biopsies immunostaining with anti-Col V peptides (**Table 1**) detected in early-SSc sera, who tested positive to anti-Col V. Interestingly, we found a strong and significant correlation to anti-Pept Col5A1(599) (r=0.930; p=0.007) and Anti-Pept Col5A1(779) (r=0.854; p=0.030) (**Supplementary Table 3**).

## Discussion

In the present study we evaluated autoimmunity to α1(V) and α2(V) chains in early-SSc patients anti-Col V positive based on the antigenicity of sera and on lung tissue specimens from SSc-ILD patients, as well as determination of the autoimmunity to five immunogenic α1(V) chain peptides and three α2(V) chain antigenic peptides in sera. We found that the reactivity between sera from early-SSc patients anti-Col V positive and SSc-ILD lung biopsies related strongly to autoimmunity to α1(V) chain, specially involving two immunogenic α1(V) chain peptides (Col5A1(1049) and Col5A1 (1439)). To our knowledge, this is the first study that explored the relationship between the anti-peptide chains of Col V antibodies present in the serum of patients with early SSc, as well as the relationship with Col V autoimmunity.

It is currently proposed that the massive exposure of the epithelial/endothelial basement membrane to autoimmunity is thought to be important because this phenomenon facilitates a marked deposition of modified interstitial collagen components (4, 5). Col V has been described extensively for more than a decade (46); however, the immunogenic role of this “minor” component of the ECM, the effect of its interaction with anti-Col V peptide autoantibodies, and the spectrum of its reactivity in the lung tissue remain poorly understood. Previous studies from our group demonstrated that Col V immunization in animals produces histological, vascular and immunological changes similar to those of human SSc (28–30). Others studies showed that not only patients with SSc present an increased expression of Col V in the skin and lung, but the anomalous Col V deposit on the skin of early-SSc patients correlates with cutaneous thickening and disease activity (20–24). Noteworthy, a generic and unique role for Col V seems unlikely because the cellular responses evoked by this collagen substrate are multiple. In fact, Col V has been associated with autoimmunity in several pathological processes, such as in the rejection of murine and human lung transplantation, hypersensitivity asthma, and atherosclerosis (8, 15–18). It is therefore not surprising that Col V is considered a sequestered antigen that can be exposed to the immune system, as a result of chronic tissue remodeling. In fact, autoimmunity to Col V had been previously described in 33% of the patients with idiopathic pulmonary fibrosis, a disease with similar pathophysiology to SSc-ILD (19).

We employed a method based in beads sensitized with bovine Col V to detect anti-Col V antibodies in early-SSc sera by flow cytometry (43). We believe that although there is the possibility that some antibodies may recognize bovine but not human Col V, the high homology between bovine and human Col V, which represents respectively 99.31% and 97.13% to Col V α1 and α2 chains, ensures that our method has a high sensitivity to detect anti-Col V antibodies in the sera of the patients. In the present study, we detected anti-Col V autoantibodies in 7 (41.18%) sera from a cohort of seventeen early-SSc patients. Surprisingly the percentage of sera from patients with early-SSc who tested positive for anti-Col V was comparable to that found in patients with idiopathic pulmonary fibrosis (19). In our study, the sera investigated came from a group of patients whose disease was characterized by very early stage of the disease. These patients do not have an exuberant clinic. By definition (EULAR Preliminary Criteria), in this disease phase the patients present basically, digital ulcers, Raynaud phenomenon, altered capillaroscopy and antinuclear antibodies or SSc specific antibodies (anti-topoisomerase 1 and anti-centromere antibody) (39, 40) (see **Supplementary Table 1**). Usually, patients at very early-stage of SSc are not submitted to skin biopsy, and in addition our patients cohort did not yet have skin thickening, and consequently the Rodnan skin score was not assessed. However, considering our previous study which increased Col V deposit was found in early-SSc skin correlated with disease activity (24), we hypothesize the α1(V) chain autoantigens could be involved in triggering autoimmunity. This hypothesis can be supported by a previous study that showed an increase in α1(V) chain gene and protein expression in early-SSc skin prior to the increased expression of α1 of the types I and III collagen and persisting until the formation of the fibrotic scar (47). In addition, a higher percentage of anti-Col V antibodies was previously detected in the early-SSc stage compared to the defined-SSc (27). From a clinical point of view, we found a strong correlation in the anti-Col V autoantibodies with the time in which the first symptom of the disease started (see **Supplementary Table 1**). Specifically, patients who had the first symptom, such as Raynaud phenomenon, for over ten years presented anti-Col V autoantibodies. This find could be explained by the presence of Col V, below the basement membrane of the vessels, which makes it exposed to chronic vascular damage. However, anti-Col V autoantibodies in early-SSc did not have association with the presence of anti-topoisomerase 1 and anti-centromere antibodies in the patients’sera (see **Supplementary Table 1**). In fact, in a previous study from our group, the association between anti-Col V and anti-topoisomerase 1 in early-SSc was also not significant (28). These findings indicate that the synthesis of anti-Col V in SSc seems to be an early event independent of other antibodies specificities. In addition, other studies have shown that the underlying mechanism for this chronology involves a primary immune response to abnormal expression of Col V. Nevertheless, to explore the results obtained in sera of the patients anti-Col V positive, as autoimmunity to Col V in the early stage of SSc, we tested sera in surgical lung biopsies from patients with defined disease, and involvement of the lung by chronic interstitial pneumonia. Because these findings correlated with a distinct explored aspect of clinical disease, we suggest the serum antibody assay to be a useful biomarker.

We also employed pulmonary biopsies from SSc-ILD patients, as antigen source to evaluate differential reactivity to α1(V) and α2(V) chains in the early-SSc sera anti-Col V positive. Another reason for this choice is that lung tissue presents a Col V isoform, similar to the skin tissue, which is composed by two α1(V) chains and one α2(V), representing 2% to 5% of the total collagen. As Col V is found embedded in the heterotypic fibrils, it has being considered a sequestered antigen (5, 11–13). Interestingly, SSc-ILD lung biopsies immunostained with the biotinylated IgG from early-SSc patients who tested positive to anti-Col V antibodies (anti-ColV IgG) showed a strong and increased green fluorescence pattern in lung compartments where Col V is usually located, such as in vessel layers, bronchi and bronchiole walls, and septal interstitium. To confirm this finding, we assessed the immune colocalization between the anti-ColV IgG of the early-SSc samples and the commercial anti-human Col V antibodies in lung biopsies from SSc-ILD patients.

Furthermore, the SSc-ILD lung biopsies immunostained with the biotinylated IgG from early-SSc patients who tested positive to anti-Col V antibodies, in which the Col V α1 chain-specific antibodies were, previously, removed by adsorption assays [anti-ColV IgG/ads-α1(V)], showed a weaker green fluorescence pattern. In contrast, when Col V α2 chain-specific antibodies were previously removed [anti-ColV IgG/ads-α2(V)], the immunostained SSc-ILD lung tissue revealed a strong green fluorescence, similar to SSc-ILD lung tissue immunestained with non-adsorbed anti-ColV IgG. Clearly, we demonstrated that when the anti-Col V IgG sample, from early SSc was adsorbed with Col V α1 chain, the antibodies specific to epitopes of the α1(V) chain were removed, and the SSc-ILD lung tissue immunostaining resulted in a weak and decreased green pattern of fluorescence in the lung compartments, in which Col V is usually located. In contrast, when adsorption was made with Col V α2 chain, SSc-ILD lung tissue showed a very strong green fluorescence, similar to unabsorbed samples indicating that anti-Col V IgG sample from early-SSc is not specific to the Col V α2 chain. In other words, the data set of these assays demonstrated that the autoimmunity to Col V is preferentially to α1(V) chain epitopes in early-SSc. Our findings agree with recent studies that show the autoimmunity to Col V in other pathologies, including lung transplant rejection and atherosclerosis, primarily targeted at the α1(V) chain (33–35, 48).

Col V is a fibrillary collagen nucleator of the Col I and III heterotypic fibrils; unlike other collagen types, Col V is highly immunogenic and considered a sequestered antigen with the potential to become an antigen when exposed to the immunologic system, which may then trigger autoimmunity (7, 10–13, 49). Taking into account all these evidences, we hypothesize that anti-α1(V)-chain autoantibodies triggered during the early phase of SSc may result from irregular fibrillogenesis or from tissue remodeling due to chronic inflammatory aggression.

In agreement with the immunofluorescence results that showed autoimmunity to the α1(V) chain in SSc, we also detected a significant expression of autoantibodies to the Col5A1(1049) and Col5A1(1439) peptides of the α1(V) chain (see **Supplementary Table 2**). Although further research is needed to assess the importance of the α2(V) chain in the pathogenesis of SSc, the dataset of this study suggests a potential role for the α1(V) chain and its Col5A1(1049) and Col5A1(1439) peptides in autoimmunity in SSc. Of note, our study is the first to investigate the anti-Col V peptides importance in SSc autoimmunity. Our findings can be relevant for the development of immunotherapeutic strategies targeting Col V as adjuvant therapies in the treatment of the SSc. In this context, we contemplate that therapies targeting the Col V segments mostly involved in SSc-triggering autoimmunity, such as the α1(V) chain and the Col5A1(1049) and Col5A1(1439) synthetic peptides, could produce a more efficient response and become a potential immunotherapeutic strategy in the treatment of SSc.

In addition, these findings could shed light on Col V intact versus degraded protein in lung tissue. Deposition of the specific antibodies to α1(V) chain, present in early-SSc patients sera, can also indicate that α1(V) chain is predominantly deposited in extracellular matrix of the SSc-ILD lung. Importantly, we found a correlation between Col V deposit in SSc-ILD lung tissue and anti-α1(V) peptides autoantibodies present in early-SSc patients sera (see **Supplementary Table 3**).

The key strength of our study is its combination of serum biomarker data and staining of disease relevant tissue. Another strength of the study is opening new immunotherapeutic strategies targeting these synthetic peptides that may be employed as adjuvant therapies in the treatment of the SSc. Actually, investigators have treated a cohort of patients with idiopathic pulmonary fibrosis who tested positive to Col V autoantibodies using oral immunotherapy with Col V (19). Their study showed this regimen was not only tolerable and safe, but also led to improved lung function (19).

One of the major limitations of our study is that we have not analyzed a large cohort because we included patients at very initial phase, which is a rare event. Most of the patients come to rheumatologist when the defined disease is manifested, even as early stage. As our first intention was determine autoimmunity to Col V, we tested the serum from patients anti-Col V proved in very early stage of SSc in defined lung tissue from SSc-ILD patients. However, it is important to emphasize that small number of cases involving patients at the very early stage of the disease, bring important information if the results obtained, mainly through statistical analysis, have been achieved through appropriate tests and carefully interpreted in order to avoid overestimation of the data. In addition, the obtained results in very early stage of SSc may establish a clinical and therapeutic protocol to prevent disease progression and thus avoid complications such as irreversible fibrosis of various organs.

## Conclusion

The successful isolation of SSc autoantibodies to Col V from early-SSc sera and applying these to the tissue of defined-SSc could refine our ability to understand the role of autoimmunity in the progression of SSc and predict new treatment strategies. The demonstration that these Col V autoantibodies recognize the α1(V) chain of the protein and react against specific α1(V) chain peptide specificities, that have been linked to other autoimmune diseases, may emerge as a useful biomarker in serum antibody assay. Although we showed the potential of this assay in small cohort of very early SSc, future validation is necessary using a similar cohort with a large set of patients to corroborate the correlations observed in our small cohort of very early SSc. The main limitation of our study is the small number of very early SSc cases used but it was minimized by the data obtained using serum and tissue. Finally, the present study is largely descriptive and exploratory, and extension of our findings are essential.

## Data Availability Statement

The raw data supporting the conclusions of this article will be made available by the authors, without undue reservation.

## Ethics Statement

The studies involving human participants were reviewed and approved by Research Ethics Committee at the Hospital das Clínicas of the University of São Paulo Medical School. The patients/participants provided their written informed consent to participate in this study.

## Author Contributions

APPV contributed to the study concept and design, acquisition of data, analysis and interpretation of data and drafting of the manuscript. LB acquisition of data, analysis and interpretation of data. ZAJQ acquisition of data, analysis and interpretation of data and statistical analysis. SC acquisition of data, analysis and interpretation of data. JTM and CF analysis and interpretation of data; statistical analysis; approval of the version to be published. CGS critical revision of the manuscript for important intellectual content. ERP selection and acquisition of tissue samples; analysis and interpretation of data. DCOA critical revision of the manuscript for important intellectual content; final approval of the version to be published. PLS analysis and interpretation of data; statistical analysis review; revising the work critically for intelectual content and final approval of the version to be published. VLC study concept and design, analysis and interpretation of data, critical revision of the manuscript for important intellectual content. WRT study concept and design, analysis and interpretation of data, critical revision of the manuscript for important intellectual content and study supervision. All authors contributed to the article and approved the submitted version.

## Funding

The research was supported by the São Paulo Research Foundation (FAPESP—Process number 2016/05617-4; Process number 2017/11865-3 and Process number 2018/20403-6).

## Conflict of Interest

The authors declare that the research was conducted in the absence of any commercial or financial relationships that could be construed as a potential conflict of interest.
