# Supplementary material for: Identification of Autoimmunity to Peptides of Collagen V α1 Chain as Newly Biomarkers of Early Stage of Systemic Sclerosis
Source: Front Immunol. 2021 Feb 12;11:604602. doi: 10.3389/fimmu.2020.604602 (PMC7907509; doi:10.3389/fimmu.2020.604602)
Supplement: Supplementary file 1 [file Table_1.pdf]

Supplementary table 1- Clinical and laboratorial features of patients with early-SSc**

	Sera Anti Col V		
	Positive	Negative	*p* value (Mann-Whitney or Fisher's exact)*
Number (%)	7 (36.8)	10 (58.8)	
Age, median (range)	54 (27-70)	49 (16-65)	0.53
Females, N (%)	7 (100)	10 (100)	-
Illness time, N (%)			
<5 years	4 (60%)	8 (80%)	0.604
>5 years	2 (40%)	2 (20%)	
Symptom time***, N (%)			
>10 years	4 (60%)	0	0.008
< 10 years	2 (20%)	10 (100%)	
Rheumatoid factor, N (%)	7 (100%)	10 (100%)	-
Telangiectasia, N (%)			
absence	6 (85.7%)	4 (40%)	0.082
presence	1 (14.3%)	6 (60%)	
Digital ulcers, N (%)			
absence	5 (71.4%)	6 (60.0%)	0.516
presence	2 (28.6%)	4 (40.0%)	
Puffy fingers			
absence	6 (87.7%)	4 (40%)	0.082
presence	1 (14.3%)	6 (60%)	
Periungueal			
capillaroscopy, N (%)	5 (71.4%)	9 (90%)	0.360
abnormal	2 (26.6%)	1 (10%)	
normal			
Anti-nuclear factor, N (%)	7 (100%)	10 (100%)	-
positive			
Anti-scleroderma 70, N (%)			
negative	6 (85.7%)	8 (80%)	0.640
positive	1 (14.3%)	2 (20%)	
Anti centriole antibody, N (%)			
negative	3 (42.9%)	5 (50%)	0.581
positive	4 (57.1%)	5 (50%)	

*Continuous variables presenting non-parametric data distribution were compared by Mann-Whitney test ($p<0.05$). Categorical variables were compared by Fisher's exact test ($p<0.05$). ** EULAR Preliminary Criteria (38,39)
***Time of the first disease symptom
