# Supplementary material for: Identification of Autoimmunity to Peptides of Collagen V α1 Chain as Newly Biomarkers of Early Stage of Systemic Sclerosis
Source: Front Immunol. 2021 Feb 12;11:604602. doi: 10.3389/fimmu.2020.604602 (PMC7907509; doi:10.3389/fimmu.2020.604602)
Supplement: Supplementary file 2 [file Table_2.pdf]

Supplementary table 2 - Detection of autoantibodies to Col V α1 and α2 chains in early-SSc sera tested positive to anti-Col V and control sera by flow cytometry

Peptides		Sera Auto-antibodies (Mean±Std. Error %**)	*p* value (Early-SSc *vs* Control)*
Col5A1(599)	Early-SSc	39.02±9.05	0.142
	Control	19.68±6.29	
Col5A1(799)	Early-SSc	22.65±7.31	0.749
	Control	23.39±5.92	
Col5A1(909)	Early-SSc	20.54±7.70	0.643
	Control	18.89±5.04	
Col5A1(1049)	Early-SSc	44.85±5.48	0.004*
	Control	14.69±3.90	
Col5A1(1439)	Early-SSc	43.74±5.25	0.006*
	Control	22.70±4.20	
Col5A2(275)	Early-SSc	11.08±2.71	0.257
	Control	7.20±3.50	
Col5A2(419)	Early-SSc	9.37±2.29	0.345
	Control	5.93±4.27	
Col5A2(1078)	Early-SSc	13.27±4.75	0.732
	Control	8.16±3.83	

*Continuous variables presenting parametric data distribution were compared by Student t-test test ($p<0.05$).

** Percentage of the fluorescent bead-bonded anti-Col V peptides in sera from early-SSc patients and healthy individuals quantified by flow cytometry.
