# Supplementary material for: Identification of Autoimmunity to Peptides of Collagen V α1 Chain as Newly Biomarkers of Early Stage of Systemic Sclerosis
Source: Front Immunol. 2021 Feb 12;11:604602. doi: 10.3389/fimmu.2020.604602 (PMC7907509; doi:10.3389/fimmu.2020.604602)
Supplement: Supplementary file 3 [file Table_3.pdf]

Supplementary Table 3 – Correlation between defined-SSc lung tissue immunostaining (Col V) and anti Col V-peptides detected in early-SSc sera (anti-ColV+)

Early-SSc sera (anti-ColV+)	Defined-SSc lung tissue immunostaining (Col V)	
Anti-Col V peptides	Correlation (r)	p*
Anti-Pept Col5A1(599)	0.930	0.007
Anti-Pept Col5A1(779)	0.854	0.030

* Kruskal-Wallis tests for comparison between defined-SSc lung tissue immunostaining and anti-Col V peptides detected in early-SSc sera. P-value<0.05 was considered significant.

r: Pearson correlation was employed to test association between continuous variables.
